# Microwave irradiation of whole soybeans in ruminant nutrition: Protein and carbohydrate metabolism *in vitro* and *in situ*

**DOI:** 10.30466/vrf.2019.35896

**Published:** 2019-12-15

**Authors:** Sara Golshan, Rasoul Pirmohammadi, Hamed Khalilvandi-Behroozyar

**Affiliations:** 1 *MSc Graduate, Department of Animal Science, Faculty of Agriculture, Urmia University, Urmia, Iran;*; 2 * Department of Animal Science, Faculty of Agriculture, Urmia University, Urmia, Iran.*

**Keywords:** Heat treatment, Metabolizable protein, Undigested crude protein

## Abstract

Whole soybeans serve as one of the main sources of protein in ruminant nutrition. Different processing methods have been employed for ruminal protein protection. The present study was conducted to determine the effects of microwave irradiation [900 W; 2, 4 and 6 min] on quality, ruminal degradability and estimated *in vitro* intestinal digestibility of availability soybean crude protein. This experiment was performed in a completely randomized design with seven treatments including control (no processing), along with 2, 4 and 6 min of microwave irradiation on whole and ground soybeans. Protein and carbohydrate fractions were determined according to Cornell Net Carbohydrate and Protein System (CNCPS). Triplicates of the samples were incubated in the rumen of three cannulated Holstein steers for up to 48 hr. Microwave irradiation increased neutral detergent insoluble nitrogen, metabolizable protein content and resulted in a lower effective rumen degradability and *in vitro* gas production. Nevertheless, longer processing time led to higher unavailable protein and carbohydrate fractions. In the main, microwave irradiation of ground samples for 4 min increased metabolizable protein content, without negative effects on protein and carbohydrate availability.

## Introduction

Whole soybeans are in use as one of the main feedstuffs for high producing dairy cows to meet protein requirements.^1^^,^^2^ However, some difficulties may be raised with the high dietary inclusion of raw soybeans such as certain anti-nutrients or anti-nutritional factors and low bypass protein.^3^ Different processing methods such as extrusion and roasting have been used to decrease rumen protein degradability using accelerating cross-linkages between free amino acids and aldehyde groups as a result of heating.^2^ Nevertheless, environmental impacts of fossil fuels, high energy and instrumental costs, difficulties in efficient processing and negative effects of extra heating either on intestinal availability of bypass protein, or increase in processing costs should be considered. Micro-wave irradiation has been reported as a quick feed processing method with simple heat control.^4^^,^^5^ Efficiency of microwave irradiation on the grains and oilseed meals was extensively investigated. ^4^^,^^5^ However, a few reports are now available about the effects of microwave irradiation on ruminal nutrient degradability of nutrients in whole oilseeds such as soybeans.^6^ The main purpose of this study is to evaluate the effects of microwave irradiation on *in situ* degradability, *in vitro* gas production and metabolizable protein parameters of the whole soybeans.

## Materials and Methods

**Sample collection, processing, and laboratory analysis. **Whole soybeans were supplied by a commercial feed processing factory and ground through a 2.00 mm screen (Wiley Mills, Thomas Scientific, Swedesboro, USA) while some remained whole. The moisture content of the sample was reached to 250 g kg^-1^ with deionized water^4^^-^^6^ and irradiation was done by a commercial microwave supplying 900 W of power, for 2, 4 and 6 min with gentle agitation in every 15 sec. This experiment was performed in a completely randomized design with seven treatments including control (no processing), along with 2, 4 and 6 min of microwave irradiation on whole and ground soybeans.

After processing, dry matter content was determined by drying at 105 ˚C overnight, and samples of different treatments were ashed by ignition in a muffle furnace (AOAC 2000, ID 942.05).^7^ The ether extract content was determined by BUCHI automated apparatus (Büchi Labortechnik AG, Flawil, Switzerland; AOAC 2000, ID 920.39).^7^ The total nitrogen (N) content was measured by the Kjeldahl method (Behr Labor-Technik GmbH, Dusseldorf, Germany) and CP was calculated as N × 6.25. The neutral and acid detergent fiber contents (NDF and ADF) were determined by using an automated Ankom Fiber apparatus (Ankom200; Ankom Technology, Macedon, USA) according to the report by Van Soest *et al*.^8^ Moreover, protein contents of NDF and ADF were measured and considered as neutral and acid detergent insoluble protein (NDICP and ADICP) respectively. In the case of CNCPS and metabolizable protein fractionation, sodium sulphite was not included in a neutral detergent solution (NDS).^9^ The chemical analysis of the samples from each of the treatments were carried out in triplicates. 

***In situ*** **degradability. **An *in situ* experiment was designed for evaluating the effects of treatments on dry matter (DM), organic matter (OM), CP, and NDF degradability, according to Vanzant *et al.*, using three ruminally fistulated Holstein steers (mean body weight, 510 ± 20.00 kg).^10^ The animals were kept individually, handled and sampled according to FASS^11^ and fed two equal meals (8:00 am and 6:00 pm) of a balanced ration (4.00 kg of Alfalfa hay, 3.00 kg of corn silage and 2.00 kg of a commercial concentrate mix) to achieve 10.00% more than maintenance energy requirements.^12^

The samples were ground to pass a 2.00 mm screen (Wiley mill) and sieved to remove particles less than 50.00 μm.^10^ Samples (5.00 g) were weighed into nylon bags (100×200 mm) with 50.00 μm pore size, to create sample size: surface area of 0.125 mg per mm^2^.^10^ Triplicates of the bags were incubated for 2, 4, 8, 12, 24 and 48 hr in the ventral rumen, just before the morning meal. Once removed, the bags were washed by agitation in cold tap water, until the rinsed water stayed clear and dried at 60.00 ˚C for 48 hr in a forced air oven and then weighed. To obtain the washing loss values, triplicates were washed with water at 39.00 ˚C for 20 min.^10^ Aliquots of bag residues were used for OM, CP and NDF determination and degradation profiles were calculated by fitting the data on the nonlinear model using the NLIN PROC of SAS statistical package (version 9.1; SAS Institute, Cary, USA).

**Fractioning of CNCPS and metabolizable protein system, intestinal protein digestibility and PDI. **The CNCPS carbohydrate fractions were determined following the procedure described by Lanzas *et al*.^13^ The A1, A2 and A3 fractions were considered to be zero and were not measured.^13^ Protein fractions of the samples were determined in triplicate, according to a standardized procedure where fraction A was determined using trichloracetic acid solution (10.00%, w/v, in water).^13^ The rumen degradable protein (RDP) and rumen undegradable protein (RUP) values were computed using National Research Council (NRC) equations.^12^ The rates of passage (kp) were considered equal to 0.05 and 0.08 percent per hour and degradation rates (kd) for B1, B2, and B3 were taken to be 150, 5.00 and 0.18% per hr, respectively.^12^

The metabolizable protein (MP) values of treated samples were calculated according to *in situ* CP degradability data as stated by Agriculture and Fisheries Research Council (AFRC).^14^ The intestinal protein digestibility was measured using three-step *in vitro* assay with 12 hr of ruminal incubation.^15^ In addition, protein dispersibility index (PDI) was measured.^16^

**Statistical Analysis. **All data except for *in situ* and gas production kinetics were analyzed with GLM procedure of SAS, with the following model:

*Y*_ij_ *= µ + G*_i_ *+ I*_j_ *+ GI*_ij_ *+e*_ij_

In the statistical model, *Y*_ij_ is the mean of the each of measurements, *µ* is the overall mean, *G*_i_ and *I*_j_ represent the fixed effects of grinding and irradiation time, respectively. *GI*_ij_ is the interaction of the main effects and *e*_ij_ is the error term_._ The least square (LS) means were adjusted and compared with Tukey and PDIFF options, respectively. Data were shown as the LS means and corresponding SEM. The *p* < 0.05 considered that differences were significant.

## Results

The effects of microwave irradiation on the chemical composition of soybeans are shown in Table 1. The organic matter, CP and total fat content of dry matter were affected neither by microwave irradiation time nor by grinding. However, the dry matter content increased with microwave irradiation compared with the control group, without significant difference between different irradiated groups. The contents of NDF, ADF and their associated nitrogenous fractions increased with longer treatment time. In addition, grinding amplified the effects of microwave irradiation on NDF, ADF, NDIN and ADIN. Also, Microwave irradiation decreased soluble protein as shown in Tables 2, 3 and 4.

**Table 1:** Chemical composition of full fat soybeans (g 100 g^-1^ DM) among different treatment groups

**Parameters**	**Control**	**M2**		**M4**		**M6**		***p *** **values**			**Contrasts, ** ***p*** ** value**			
		**M**	**W**	**M**	**W**	**M**	**W**	**SEM**	**Mic**	**Mill**	**Control vs. Processed**	**M2 vs. M4**	**M4 vs. M6**	**M2 vs. M6**
**DM**	89.27	94.10	93.85	95.09	92.89	96.01	94.24	1.12	0.0069	0.65	0.001	0.9856	0.3291	0.3377
**OM**	93.91	94.33	92.79	93.67	92.31	93.29	92.55	1.13	0.9172	0.81	0.7456	0.619	0.9497	0.5759
**CP**	36.08	36.78	36.10	36.55	35.66	36.36	35.89	0.44	0.7098	0.48	0.4261	0.4529	0.9526	0.4885
**EE**	18.19	18.60	18.44	18.34	18.42	18.47	18.33	0.22	0.9096	0.57	0.8113	0.5333	0.9303	0.5913
**NDF**	18.12	19.46	18.29	20.61	21.15	25.30	23.66	0.23	<0.0001	0.02	<0.0001	<0.0001	<0.0001	<0.0001
**ADF**	14.16	14.53	14.28	15.55	14.83	15.37	15.25	0.17	<0.0001	0.21	0.0009	0.0004	0.5151	0.0001
**NDICP**	7.10	11.33	10.66	11.91	11.31	13.37	12.69	0.12	<0.0001	0.00	<0.0001	0.0001	<0.0001	<0.0001
**ADICP**	4.86	5.17	5.02	5.23	5.11	5.38	5.18	0.06	0.0004	0.08	0.0002	0.2669	0.0824	0.0084

DM: Dry matter; OM: Organic matter; CP: Crude protein; EE: Ether extract; NDF: Neutral detergent fiber; ADF: Acid detergent fiber; NDICP: Neutral detergent insoluble crude protein; ADICP: Acid detergent insoluble crude protein; M2: Microwave 2 min; M4: Microwave 4 min; M6: Microwave 6 min; M: Milled; W: Whole; Mic: Microwave irradiation; Mill: Milling.

The effects of microwave irradiation on ruminal degradation parameters of DM, OM, CP, and NDF are shown in Table 2. In general, microwave irradiation declined the ultimate disappearance of the measured nutrients from the nylon bags. Irradiation of the ground samples did not develop a significant difference in DM degradation kinetics compared with the whole samples, but in the case of OM, CP, and NDF, the ground samples had lower ruminal degradability. Unlike CP, degradation kinetics for NDF did not show linear responses to heating time. In initial incubation times, NDF degradability was higher for short time irradiated samples.

As shown in Table 2, microwave irradiation for 2 min increased the potentially degradable fraction of DM, OM, and CP. On the other hand, a longer irradiation time caused lower fraction A and B, and increased indigestible fraction C.

**Table 2:** Ruminal nutrient degradability parameters of full fat soybeans among different treatment groups

**Parameters**	**Control**	**M2**		**M4**		**M6**		***p *** **values**			**Contrasts, ** ***p*** ** value**			
		**M**	**W**	**M**	**W**	**M**	**W**	**SEM**	**Mic**	**Mill**	**Control vs. Processed**	**M2 vs. M4**	**M4 vs. M6**	**M2 vs. M6**
***Dry matter***														
**A (%)**	45.43	21.32	35.93	23.05	30.27	22.33	29.15	1.94	<0.0001	0.01	<0.0001	0.0786	0.0748	0.9787
**B (%)**	49.26	77.21	56.90	56.82	46.24	47.87	54.52	3.71	0.0009	0.00	0.0298	0.0009	0.9292	0.0008
**UD (%)**	5.31	1.47	7.17	20.13	23.49	29.8	16.33	1.30	<0.0001	0.04	<0.0001	0.0174	0.0003	<0.0001
**C per hr (%)**	0.11	0.09	0.10	0.06	0.11	0.05	0.07	0.06	0.5571	0.33	0.7731	0.9887	0.2358	0.2307
**ED 0.03 (%)**	79.77	70.61	72.39	65.74	64.63	56.46	66.06	1.66	<0.0001	0.04	<0.0001	0.0019	0.0332	<0.0001
**ED 0.05 (%)**	73.11	63.58	62.53	60.01	57.98	50.50	59.27	1.17	<0.0001	0.10	<0.0001	0.0038	0.0035	<0.0001
**ED 0.08 (%)**	66.01	56.17	52.83	54.50	50.84	44.12	52.50	0.88	<0.0001	0.59	<0.0001	0.0567	0.0002	<0.0001
***Organic matter***														
**A %**	42.83	22.94	30.14	24.95	36.33	18.65	33.94	2.06	<0.0001	0.579	<0.0001	0.0663	0.0532	0.9066
**B %**	51.59	74.40	57.94	57.85	46.95	55.16	49.04	3.97	0.0062	0.03	0.1252	0.0038	0.9398	0.0033
**UD %**	5.58	2.66	11.92	17.2	16.72	26.19	17.02	1.20	<0.0001	<0.0001	<0.0001	0.2773	0.0011	<0.0001
**C per hr (%)**	0.13	0.11	0.09	0.07	0.08	0.09	0.13	0.02	0.4312	0.27	0.1693	0.7677	0.5611	0.3852
**ED 0.03 (%)**	81.31	72.02	73.45	67.25	67.79	58.63	68.52	1.44	<0.0001	0.01	<0.0001	0.0029	0.0166	<0.0001
**ED 0.05 (%)**	75.19	65.03	63.91	61.44	61.01	52.12	62.07	0.99	<0.0001	0.01	<0.0001	0.0057	0.001	<0.0001
**ED 0.08 (%)**	68.42	57.57	54.25	55.78	53.57	45.23	55.18	0.732	<0.0001	0.05	<0.0001	0.1135	<0.0001	<0.0001
***Crude protein***														
**A (%)**	48.24	35.31	37.91	30.68	32.34	28.75	34.83	2.59	0.0063	<0.0001	0.0572	0.0019	0.0906	0.0656
**B (%)**	46.47	58.22	58.03	62.09	55.68	58.85	52.70	8.66	0.0063	0.04	0.0454	0.0435	0.1525	0.0022
**UD (%)**	5.29	6.47	4.06	7.23	11.98	12.4	12.47	1.54	<0.0001	0.03	<0.0001	0.0481	<0.0001	<0.0001
**C per hr (%)**	0.06	0.04	0.04	0.04	0.04	0.03	0.04	0.017	0.0465	0.06	0.1112	0.2438	0.0167	0.1557
**ED 0.03 (%)**	70.40	62.14	66.68	63.59	64.15	45.22	57.64	2.32	0.0041	<0.0001	0.0631	0.1498	0.0227	0.0011
**ED 0.05 (%)**	61.97	53.53	58.71	56.43	56.73	39.58	54.79	1.40	0.0069	<0.0001	0.2105	0.4734	0.0076	0.0018
**ED 0.08 (%)**	54.57	44.71	52.24	50.21	53.99	33.37	52.31	0.71	0.0012	<0.0001	0.69	0.0387	0.0001	0.0096
***Neutral detergent fiber***														
**A (%)**	39.93	40.85	41.31	47.46	41.03	31.97	40.73	1.85	0.0033	0.26	0.6485	0.0017	0.0008	0.6993
**B (%)**	54.37	48.31	46.72	36.83	40.68	41.91	43.25	4.70	0.0033	0.06	0.0539	0.0014	0.43	0.0068
**UD (%)**	5.70	10.84	11.97	15.71	18.29	26.12	16.02	3.56	<0.0001	0.04	<0.112	0. 374	0.345	<0.0001
**C per hr (%)**	0.11	0.09	0.12	0.08	0.13	0.09	0.09	0.18	0.8097	0.45	0.8788	0.4583	0.8927	0.3833
**ED 0.03 (%)**	77.15	73.38	76.29	70.01	69.92	62.17	69.68	1.49	<0.0001	0.02	<0.0001	0.0056	0.017	<0.0001
**ED 0.05 (%)**	70.07	67.42	67.69	65.36	64.81	57.29	64.16	1.02	<0.0001	0.03	<0.0001	0.0298	0.0008	<0.0001
**ED 0.08 (%)**	62.72	61.25	59.59	61.01	59.36	52.21	58.65	0.71	<0.0001	0.14	<0.0001	0.744	<0.0001	<0.0001

A: Soluble fraction; B: Potentially degradable fraction; UD: Undegradable fraction; C: Degradation rate; ED: Effective degradability in different rumen outflow rates; M: Milled; W: Whole; Mic: Microwave irradiation; Mill: Milling; M2: Microwave 2 min; M4: Microwave 4 min; M6: Microwave 6 min; M: Milled; W: Whole; Mic: Microwave irradiation; Mill: Milling.

**Table 3:** Metabolizable protein parameters of full fat soybeans (g 100 g^-1^ CP) among different treatment groups

**Parameters**	**Control**	**M2**		**M4**		**M6**		***p *** **values**			**Contrasts, ** ***p*** ** value**			
		**M**	**W**	**M**	**W**	**M**	**W**	**SEM**	**Mic**	**Mill**	**Control vs. Processed**	**M2 vs. M4**	**M4 vs. M6**	**M2 vs. M6**
**QDP**	47.99	34.89	38.50	30.01	32.31	28.47	34.46	0.69	<0.0001	<0.0001	<0.0001	<0.0001	0.6905	<0.0001
**SDP**	34.64	32.12	26.43	28.27	24.09	21.85	23.32	0.52	<0.0001	<0.0002	<0.0001	<0.0002	<0.0003	<0.0004
**RDP**	82.63	67.01	64.93	58.29	56.41	50.33	57.72	1.21	<0.0001	0.8351	<0.0001	<0.0001	0.0149	<0.0001
**ERDP**	73.03	60.03	57.23	52.29	49.94	44.63	50.84	1.07	<0.0001	0.6696	<0.0001	<0.0001	0.0064	<0.0001
**UDP**	16.91	32.75	36.27	42.89	44.31	49.45	41.67	0.92	<0.0001	0.3909	<0.0001	<0.0001	0.0496	<0.0001
**DUP**	13.72	27.97	31.19	36.95	38.32	42.84	35.91	0.79	<0.0001	0.4041	<0.0001	<0.0001	0.045	<0.0001
**MP**	60.46	67.35	67.32	73.13	70.75	71.74	68.81	1.58	<0.0001	0.4544	<0.0001	0.0102	0.3081	0.0818

QDP: Quickly degradable protein; SDP: Slowly degradable protein; RDP: Rumen degradable protein; ERDP: Effective rumen degradable protein; UDP: Undegradable protein; DUP: Digestible undegradable protein; MP: Metabolizable protein; M2: Microwave 2 min; M4: Microwave 4 min; M6: Microwave 6 min; M: Milled; W: Whole; Mic: Microwave irradiation; Mill: Milling.

**Table 4:** The CNCPS protein fractions and RDP and RUP content of full fat soybeans (g 100 g^-1^ CP) among different treatment groups

**Parameters**	**Control**	**M2**		**M4**		**M6**		***p *** **values**			**Contrasts, ** ***p*** ** value**			
		**M**	**W**	**M**	**W**	**M**	**W**	**SEM**	**Mic**	**Mill**	**Control vs. Processed**	**M2 vs. M4**	**M4 vs. M6**	**M2 vs. M6**
**A**	22.89	17.06	17.88	14.32	16.56	10.81	13.73	0.382	<0.0001	<0.0001	<0.0001	<0.0002	<0.0003	<0.0004
**B**	65.47	73.16	71.31	79.65	73.60	76.84	74.21	1.674	<0.0001	0.0923	<0.0001	0.0185	0.5205	0.067
**B1**	45.17	10.24	12.99	9.71	11.25	7.29	9.79	0.396	<0.0001	<0.0001	<0.0001	0.0111	0.0002	<0.0001
**B2**	14.19	49.65	45.71	53.95	47.84	52.38	47.85	1.066	<0.0001	0.0002	<0.0001	0.0082	0.4765	0.0363
**B3**	6.11	13.27	12.60	15.99	14.51	17.17	16.56	0.319	<0.0001	0.0119	<0.0001	<0.0001	0.0001	<0.0001
**C**	11.64	11.63	11.54	12.36	11.70	13.06	12.54	0.268	0.0021	0.2466	0.1761	0.1177	0.0107	0.0003
**RDP 0.08**	71.36	46.17	48.08	44.64	45.96	38.25	41.79	1.103	<0.0001	0.0107	<0.0001	0.1174	0.0002	<0.0001
**RUP 0.08**	28.64	55.68	52.66	61.69	55.90	62.46	58.68	1.245	<0.0001	0.0043	<0.0001	0.0019	0.1732	<0.0001
**Digestibility**	88.23	88.87	88.71	87.43	85.80	85.76	86.09	0.994	0.0308	0.6753	0.1419	0.044	0.4939	0.0107
**PDI %**	53.11	13.39	14.96	12.28	12.75	9.33	10.32	0.413	<0.0001	0.0103	<0.0001	0.001	<0.0001	<0.0001

A: Non protein nitrogen soluble fraction; B1: Soluble true protein; B2: Potentially rumen degradable true protein; B3: Rumen undegradable fraction; C: Indigestible fraction, RDP: Rumen degradable protein in rumen outflow rate of 0.08, RUP: Rumen undegradable protein in rumen outflow rate of 0.08; PDI: Protein dispersibility index; M2: Microwave 2 min; M4: Microwave 4 min; M6: Microwave 6 min; M: Milled; W: Whole; Mic: Microwave irradiation; Mill: Milling.

The degradation rate was not affected by the treatments, except for CP. Processing of the soybeans with microwaves significantly increased rumen undegradable protein fraction subjected to irradiation time.

The effects of microwave irradiation on metabolizable protein profile are shown in Table 3. Irradiation for 2 and 4 min significantly increased rumen undegradable protein (UDP), digestible undegradable protein (DUP) and metabolizable protein (MP), but longer processing led to a worse ratio of MP and DUP to UDP.

The CNCPS protein fractions, PDI and the intestinal protein digestibility were represented in Table 4. Fraction A and also total B fractions were affected by the treatments, but the unavailable protein was not affected. Microwave irradiation reduced calculated RDP and caused higher RUP, as a function of processing time. The processing for 6 min, decreased PDI to a desirable point but negatively affected intestinal digestibility.

Microwave irradiation significantly changed protein dispersibility index (PDI) *in vitro* but intestinal protein digestibility was not affected (Table 4). The measured PDI was 13.39, 12.28 and 9.33 for ground and 14.96, 12.75 and 10.32 for whole samples, respectively after 2, 4 and 6 min of irradiation.

## Discussion

As shown in Table 1, microwave irradiation increased the DM content of the samples, which may indicate microwave ability to decrease the moisture-holding capacity of the soybeans. Another Similar study has shown the higher DM content for microwave irradiated feeds.^17^ The crude protein, OM and ether extract content of the samples were not affected by microwave irradiation, as the results were reported for cottonseed.^17^^,^^18^ Nevertheless, contradictory results were reported by Thongprajukaew *et al.* regarding soybean meal.^19^ This inconsistency may be due to the fatty acid content, irradiation time and applied temperature. Fundamentally, irradiation can induce the release of unsaturated fatty acids, makes oxidative conditions and may cause the formation of secondary oxidative products.^20^

The major fiber fractions measured as NDF and ADF were affected by heating and NDF was increased as a function of irradiation time. Increased crude fiber and NDF content in heated samples had been reported previously.^21^^-^^23^ Instead, researchers reported no changes or even reduced fiber content in the heat-treated feed samples.^16^^,^^23^

 Microwave irradiation decreased soluble protein as shown in Tables 2, 3 and 4. This effect is comparable to the findings of soluble protein reduction of camelina seeds (52.73 to 20.41% CP), soybeans (43.38 to 11.35% CP) and flaxseeds (51.88 to 8.82% CP) as a result of moist heating at 120 ˚C for 60 min. Recovered nitrogen in NDF and ADF increased with microwave irradiation and extended treatment, led to greater values. Comparable results were reported by researchers who observed the same results in the higher cell wall bound CP with conventional heating methods.^16^ The higher cell wall associated CP could be related to Maillard reactions.^8^^,^^24^

In the present study, NDCIP was increased more than ADICP, which was in agreement with the previous findings.^21^^,^^23^ A decrease in the soluble protein of the samples (Tables 2, 3 and 4) in this study, can be used to explain more increase in NDCIP than ADCIP in the heated samples. Commonly, heat treatment may increase NDICP, but its effect on ADICP can be variable, depending on the differences in ADF and NDF content and composition, feed CP content and fractions, soluble protein content, along with the type and duration of heating.^21^ Some reports revealed the recovery of less soluble and heat denatured proteins in NDF, but, protein recovery in ADF fraction may require higher heat input than what is need for increase in NDICP^22^^,^^ 24^

*In vitro* and *in situ* experiments have shown that the rate and the extent of ruminal degradation of protein may be reduced by conventional heat treatment such as roasting, extruding or moist heating techniques.^24^ When degradation rate (K_d_) remains unchanged, the lower effective ruminal degradability might be related to the lower soluble and the higher potentially degradable fractions of the CP in the treated samples (Tables 2 and 4). In contrast to the present study, many reports revealed the lower K_d_ for microwave treated feed samples.^4^^,^^5^^,^^25^ This inconsistency may be due to the lower K_d_ of untreated samples in this study compared with control groups reported in the literature. Different factors such as pre-treatment processing, heating extent, and duration, amount of moisture, applied pressure, energy input and finally plant variety and growing conditions, may explain the miscellaneous results.^12^^,^^23^ Protein degradability was affected more than DM, OM and NDF parameters by microwave irradiation. This may be attributed to the non-thermal effects of microwave irradiation in the excitation of the protein reconstruction.^26^ The mechanisms behind the protection of protein against the ruminal degradation in the heat-treated feedstuffs are complex. However, the development of chemical reactions during heat processing can be used to explain part of the results.^27^^,^^28^ Blockage of the reactive sites for microbial proteolytic enzymes as a result of heating was suggested as an explanation for the reduced ruminal CP and DM degradability.^29^ Besides, Van Soest declared an increase in CP escape through heating as a result of CP denaturation and reduction in solubility and degradation rate.^23^ Moreover, cross-linking of the polypeptide chains, and protein aggregation,^30^^, ^^31^ unfolding of protein structures and increasing the surface hydrophobicity by exposing non-polar groups,^32^ electrostatic interactions and formation of disulfide bonds^33,34^ may be aid to explain. In line with *in situ* results, microwave irradiation decreased *in vitro* ruminal gas production which may be due to the unavailable nutrients created by heating.^4^^,^^5^^,^^25^

As shown in Table 3, microwave irradiation significantly increased the metabolizable protein content, mainly by reduction of effective rumen degradable protein, and unaffected DUP to UDP ratio. These results are in line with rumen protein degradability data (Table 3). Similarly, DUP:UDP ratios between control and treated soybeans were in line with unchanged *in vitro* intestinal protein digestibility as shown in Table 4 and slightly higher unavailable fraction as measured *in situ.* Carbohydrate fractions were less affected by the treatments than proteins. Microwave irradiation had no effects on starch, soluble and digestible fiber fractions, but sugar content decreased and indigestible fiber fraction increased with 6 min of microwave irradiation. It is possible that the higher temperature resulted in the reaction between sugars and amino acids, and then the sugars were recovered in acid detergent insoluble fraction. 

As shown in Table 4, microwave irradiation up to 4 min did not significantly change unavailable protein fraction in the cornel system, which could be possible support for MP system results. Microwave irradiation generally diminished soluble protein (A and B_1_) and increased B_2_ and B_3_ in CNCPS fractionation analogous to *in situ* degradability and MP system. This study is the first to report the effects of microwave irradiation on protein quality of soybeans, but comparable results can be found in the literature about the other heating methods.^16^^,^^21^^,^^35^^,^^36^ Extensive heating around 100 ˚C was reported to cause the loss of labile amino acids such as cysteine and lysine.^37^^,^^38^

Protein denaturation due to heating served as the main factor contributed to lower protein solubility after applying the heat treatments.^23^ The increase in fraction B2 in microwave treated samples may account in part for losses seen in the fraction B1. This shift in fractions B, accompanied by a reduction in fraction A can cause slower rumen degradability and then lower effective rumen degradability for treated samples compared to control samples (Table 2). Reduction in the crude protein solubility and subsequently buffer solubility and ruminal degradability as a result of heating is well known and frequently reported.^36^^,^^39^ Additionally, changes in the percentage of secondary protein structures such as a reduction in α-helixes and multiplication of β-sheets could be considered as a causal factor in heat-treated samples.^40^ A high proportion of heat-sensitive proteins (globulins and albumins) and amino acids (cysteine and lysine) in soybean made it prone to heating effects.^23^

At least a part of increased B3 fraction can be related to the increased NDICP and no change in ADICP. Increased NDICP is an accepted phenomenon consequent to moderate heating of high protein content of feeds due to the protein denaturation and recovery of denatured and less soluble proteins in NDF.^35^^,^^36^^,^^41^^,^^42^ However, the higher temperature or heating time may be required for amino acid and sugar complexes to be made and recovered as ADICP.^23^ The Effect of heating on ADICP seems to be inconsistently higher ^43^, and unchanged ^35^ and even lower amounts ^16^ of ADIN has been reported for heat-treated feed samples compared to the control group. The availability of reducing sugars, free reactive amino acids, pH, as well as heating extent and durability can affect net results of heating on protein recovery in acid detergent insoluble fraction.^23^

In the present study, microwave irradiation up to 4 min did not significantly change ADICP. These results can be interpreted with reported data for *in situ* degradability and MP (Tables 2 and 3). It can be concluded that the processing condition used in the present study is not so harsh to generate unavailable protein. This conclusion can be further supported by unchanged *in vitro* intestinal digestibility of crude protein (Table 4). It seems that microwave had better performance in the case of ground samples to change protein degradability and metabolizability characteristics. This is the first report about the effects of sample pretreatment on microwave irradiation efficiency and nutrient availability of microwave irradiated samples. Rafiee-Yarandi *et al*. revealed that fine grinding of soybeans before roasting was resulted in higher CP availability compared with coarse or whole samples, measured as NDICP, ADICP, and CNCPS protein fractions, but there was no explanation for the results.^16^ Carbohydrate fractions did not change over microwave irradiation unless the C (unavailable fraction) increased as a result of irradiation above 2 min (1.68 g 100^-1^ for control versus 1.72 and 2.12 for 4 and 6 min irradiated samples, respectively).

Microwave irradiation significantly changed protein dispersibility index (PDI) *in vitro* but intestinal protein digestibility was not affected (Table 4). The measured PDI was 13.39, 12.28 and 9.33 for ground and 14.96, 12.75 and 10.32 for whole samples, respectively after 2, 4 and 6 min of irradiation. Particle size reduction before processing, roasting time and the temperature has been reported that they significantly affect the dispersibility index of soybean protein.^16 ^The ideal range for PDI has been reported to be 9-11, while values between 11 and 14 specified marginal heating. Moreover, suboptimal and over-heated samples will have PDI more than 14 and lower than 9, respectively. The dispersibility index decreased linearly with increased processing time and was in the appropriate range, except for whole beans irradiated for 2 min.

 Relevant reports can be found in the literature confirming the lower PDI for higher temperatures or longer heating duration in the case of extruded or roasted feeds.^43-47 ^The lower PDI for ground irradiated samples in the present study can be attributed to the greater processing extent due to the higher ability of microwaves to penetrate the kernel of the samples with excluding the seed coat. However, the lower PDI for whole compared with ground roasted soybeans has been reported.^16^ In a recent study, researchers reported no significant correlation between PDI and unavailable protein fraction in CNCPS.^16^ Nevertheless, a significant negative correlation was reported between PDI and rumen undegradable protein.^44^^-^^47^ Conflicting results between experiments could be attributed to the different heating methods. 

Microwave irradiation decreased ruminal protein degradability. Notwithstanding longer irradiation time was accompanied by higher unavailable nutrients, and stunted processing resulted in suboptimal results. Unchanged ADICP and a simultaneous increase in NDICP have been proven as the important features of a non-destructive and efficient processing to maximize digestible undegradable protein.^22^^,^^42^^,^^43^ Thus, microwave irradiation can serve as an optimal, efficient and affordable processing method for feed.

The optimal condition for microwave irradiation in this study could be defined as irradiation of coarse ground samples up to 4 min with the microwave. The results of this experiment showed that microwave irradiation can serve as an optimal processing method to bypass more protein from the rumen in the case of soybeans without negative effects on intestinal availability. However, it seems that microwave had better performance in the case of ground samples to change protein metabolizability characteristics. More research could be recommended in the case of pre-treatments, processing time and to explore potential differences between different oilseeds. 
